# The association of cultural and contextual factors with social contact avoidance during the COVID-19 pandemic

**DOI:** 10.1371/journal.pone.0261858

**Published:** 2021-12-28

**Authors:** Wolfgang Messner

**Affiliations:** Darla Moore School of Business, University of South Carolina, Columbia, SC, United States of America; Shahjalal University of Science and Technology, BANGLADESH

## Abstract

As a first line of defense to the COVID-19 pandemic in 2020, people reduced social contacts to avoid pathogen exposure. Using a panel of countries, this research suggests that this was amplified in societies characterized by high social support and future orientation. People reacted more strongly in dense environments; government orders had more effect in high power distance societies. Conversely, a focus on accomplishments was associated with lower changes. Understanding people’s actual behaviors in response to health threats across societies is of great importance for epidemiology, public health, international business, and for the functioning of humanity as a whole.

## Introduction

First reports of a pneumonia of unknown etiology emerged in Wuhan, China, on December 31, 2019. The extremely contagious virus was identified as severe acute respiratory syndrome coronavirus 2 (SARS-CoV-2), causing the disease COVID-19. It spread quickly with regional differences in the outbreak rate between countries [1, 2] and within countries [3, 4]. By the end of 2020, COVID-19 infected 72.4 million people and caused 1.8 million deaths worldwide [5]. The pandemic embodied all characteristics of a disaster and mass emergency [6, 7]. But the actual threat to be infected was not from people per se, but rather from the pathogens they potentially harbored. Because pathogens are invisible and symptoms often hidden (so-called asymptomatic carriers) or delayed (incubation period), it was difficult to identify actual pathogen carriers. In the absence of effective countermeasures (vaccines) or treatments (medicines), the only solution was for individuals to avoid social contact, and for governments to strengthen aversion to social contact through rules and regulations [8]. The threat of COVID-19 differed from threats posed by other common illnesses, such as influenza, which have widely available treatments or vaccines, and pose low levels of risk to otherwise healthy individuals [9]. In the past decades, we–fortunately–did not witness the outbreak of an infectious disease at such a global scale. Our research possibilities into cultural aspects of behavioral changes as a response to health threats were thus rather limited. Even so, “epidemics are not an esoteric subfield for the interested specialist but instead are a major part of the ‘big picture’ of historical change and development. Infectious diseases […] are as important to understanding societal development as economic crises, wars, revolutions, and demographic change” [10]. Past epidemics, such as the bubonic plague that devastated London in the middle of the seventeenth century or the Spanish influenza of 1918, always resulted in significant changes in society reflected in the arts and literature, realms of science and technology, government and public health practices [11]. This relates well into ideas about the evolution of social life, called the civilizing process [12]. Also, “there is no question that the COVID-19 pandemic […] created a unique set of pressures for the conduct of international business and the pursuit of globalization” [13]. Over the course of the human evolutionary history, the management of infectious diseases has been one of the most enduring and fundamental problems [14]; the intellectual challenge to appreciate what was at stake during the COVID-19 pandemic certainly counts as a grand challenge [15]. Putting the matter more narrowly, to what extent did cultural and contextual factors amplify social contact avoidance? And how did government response policies contribute in different environments?

Because extant disaster research maintains that “community-documented behaviors are a stronger test of the stress-reaction effect of disasters than are subjective, after-the-fact self-reports” [16], I use country-level longitudinal mobility change data at places of residence made publicly available by Google for 135 countries, from February 2020 to January 2021. Comparing changes to a pre-pandemic baseline is an approximation for the extent of contact avoidance outside of the home. With a longitudinal multilevel model, I then regress these changes on the pandemic threat level and the stringency of government response policies. At the ecological level, I examine the influence of national culture and other contextual factors for 39 countries. At the time of writing in 2021, COVID-19 was still too new to assess its ultimate impact, but its broad contours were sufficiently clear to allow this study. The question was, whether, after COVID-19 (hopefully) abated, the world, science, and governments, would return to complacency as they have done with past epidemics, such as outbreaks of avian flu, severe acute respiratory syndrome (SARS), swine flu (influenza A, H1N1), Middle East respiratory syndrome (MERS), Marburg, and Ebola between 2003 and 2016 [10]–or whether they would use data and insight generated through studies such as this one for a sustainable and long-term assessment of the health challenges, and get organized to face them heads-on. In parallel, businesses also needed to understand how the pandemic changed their customers’ behavior, and what they could learn from COVID-19 for future global disasters.

The article proceeds as follows. I start with an overview of the extant literature with respect to terror management theory, the terror management health model, the behavioral immune system, and cross-cultural aspects of human behavior. Next, I theorize potential connections between national culture and other contextual factors with social contact avoidance, caused by changes in the pandemic’s perceived threat level and mobility restrictions imposed by governments. In the materials and methods section, I delineate the variables, describe the data, and present the research model. Subsequently, I construct the multilevel model, and test the research hypotheses. I describe implications for cross-cultural psychology, public health policy, and international business; I conclude with a detailed discussion of the study’s inherent limitations.

## Research context

### Terror management theory and terror management health model

Terror management theory offers predictions for how people behave in response to fear associated with mortality [17]. Grounded in evolutionary theory, it first proposes that humans exhibit a biological predisposition toward self-preservation and reproduction, which they share with other life forms. Second, it acknowledges that humans are unique in their use of conscious thought processes. In novel circumstances, humans are thus able to delay behavior in order to ponder alternative responses, “and imagine that which does not yet exist, and transform the physical universe accordingly” [18]. Over the last three decades, hundreds of studies have empirically illustrated and supported this mortality salience hypothesis [19].

In turn, the terror management health model applies these ideas to health behaviors and decisions [20]. It is particularly relevant for infectious diseases, because, in the 20^th^ century, the annual mortality rate from infectious diseases outpaced the annual mortality rate from all wars worldwide combined [14]; and on January 19, 2021, the milestone was reached when more Americans had succumbed to COVID-19 than the number of US troops killed during World War II (405399 as per the Department of Veterans Affairs [21]). Proximal defenses to health threats center around two types of self-preservation efforts. First, perceived vulnerability can be reduced through denying susceptibility to a disease, avoiding detection through tests, or suppressing its deadliness. Such denial and suppression responses were displayed publicly when UK Prime Minister Boris Johnson shook hands with COVID-19 patients [22], or when Brazilian President Jair Bolsonaro and US President Donald Trump compared COVID-19 to the seasonal flu [23]. Second, by engaging in health-promoting behaviors aimed at reducing the risk of infection, such as ensuring proper hand hygiene, wearing personal protective equipment (e.g., face masks), or minimizing social contact [24, 25]. For example, the British digital marketing agency Reboot reminded its employees to wash their hands every thirty minutes with an egg timer [26]. Just a little later in the course of the pandemic, governments banned non-essential travel and closed borders [27]. Feeling anxious and engaging in an increased number of such safety behaviors are adaptive responses to a real threat, namely high numbers of infections and deaths [9]. As such, the terror management health model mirrors behavioral health responses that are also investigated in the context of research on personally relevant, fear-arousing threats [28, 29]. However, “it is not yet known […] what factors predispose an individual to high levels of anxiety during a pandemic” [9].

### Behavioral immune system

Related to the terror management health model, an emerging literature within evolutionary social psychology has highlighted that humans possess a system of specialized cognitive, behavioral, and emotional mechanisms, by which they manage pathogenic threats. This so-called behavioral immune system is a first line of defense to minimize exposure to dangerous viruses and bacteria, ahead of a response by the more metabolically costly physiological immune system [30–33]. Moreover, a response by the latter is only effective after infection, while the former as a complementary psychological mechanism can mitigate contact with pathogen carriers in the first place [14]. Extant research on the behavioral immune system reveals that pathogen-avoidance reactions include attention, movements, preferences, and prejudicial attitudes toward people as potential pathogen carriers [14, 34–39]. In this research, I examine the extent of social contact avoidance as one such movement in response to perceived risk of exposure to SARS-CoV-2, putting a special focus on the amplification aspects of cultural and other contextual factors. To mitigate feelings of discomfort, people will seek greater social distance from their interaction partners [40]. I thereby aim to analyze an important observation in extant empirical COVID-19 research, which documented individuals reporting high safety compliance without endorsing pandemic anxiety [41]. Because there are individual differences in the sensitivity of the behavioral immune system [42], “there are likely factors (i.e. social desirability) other than fear of contamination that drive safety behavior usage” [9].

### Cross-cultural aspects

It is well-documented that human behaviors reflect the prevailing values of the culture to which they belong [43–45]; a country’s culture shapes its people’s perceptions, dispositions, and behaviors [46]. Logic and the mode of thinking are also cultural phenomena [47]. Moreover, goal and action identification theories [48, 49] suggest that human actions are partly determined by underlying goals that may operate in a given context [50].

Humans’ awareness of death creates the potential for debilitating terror, managed through humanly created cultural belief systems. These systems are shared within social groups, and are the foundation for minimizing anxiety engendered by the awareness of death [18]. Thus, different cultures react to different threats in specific ways, with the ultimate aim of restoring freedom [51]. This difference is grounded in varying expectations of control and choice learned through socialization [52]. Accordingly, individuals in all cultures will react to a pandemic caused by a contagious pathogen with contact avoidance, but its extent will be different. Therefore, my aim in this study is to examine whether national culture affected the extent of social contact avoidance during the COVID-19 pandemic. In doing so, I follow the tradition of *gestalt*-oriented social psychologist Kurt Lewin (1890–1947), who conceptualized human actions or behavior as a function of both the person and the environment the person is in. Regarding the perspective of the person, parts of psychology and social science work with the assumption of common features in different cultural groups [53]. In a universalist approach, groups of people are compared in order to work out their homogeneous core in the form of “researchable features” [54]. Regarding the environment, anthropology maintains that no cultures are alike. Consequently, cross-cultural generalizations are not warranted, “each culture must be considered as a unique configuration” [53], and hypotheses about causes and effects need to account for cultural specificities [55–58].

### Activation of the behavioral immune system

The behavioral immune system helps people to deploy mechanisms aimed at avoiding contact with harmful pathogens. Because other humans are transmission vectors, an increase in the overall pandemic threat level elevates people’s reluctance to enter social situations [30]. As viruses and bacteria are largely invisible and people can be asymptomatic carriers, it is difficult to reliably detect infectious persons. Hence, people will find simpler ways of avoiding social contacts. I propose that the degree to which the behavioral immune system triggers contact avoidance generally depends on the threat level present in the environment. I posit the following introductory hypothesis:

**H1**: When the pandemic threat level is high, people generally avoid more social contacts.

During the COVID-19 pandemic, governments around the world instigated countermeasures to reduce domestic transmission of the virus and block the entry (or re-entry) of foreign risk factors. For example, in South Korea, the government prepared strict guidelines as early as March 2020 towards the usage of public spaces and facilities that normally accommodate many people; even the start of the spring semester for daycare centers and schools was delayed [59]. Later in the course of the pandemic, the University of California, Berkeley, even banned their residential students’ outdoor exercise [60]. Hence, I expect rules and regulations introduced by governments and other regulatory bodies to coerce people into social contact avoidance. More formally:

**H2**: Mobility restrictions introduced by authorities are concomitant with people avoiding more social contacts.

Adaptive responses, such as frequent handwashing and wearing personal protective equipment, are relatively easily actionable. But mandates and recommendations to socially distance and stay at home are far more difficult to accomplish effectively [28], because they can have far-reaching implications for the functioning of society. Hence, motivations underlying such mitigating behaviors to counter pandemic threat must also consider cultural and other contextual factors. In the following, I group these factors into five areas: (1) Social support; (2) Time orientation; (3) Focus on accomplishments; (4) Environmental psychology; and (5) Acceptance of rules.

### Social support

Adherence to social norms is an important collective response to a communal crisis [61]. As a cultural value, collectivism emphasizes ties between people. Because they value relational harmony, collectivists tend to be interdependent, recognize their duties and obligations to others [62], interact in a cooperative mode [63], and generally conform and adapt to fit the environment in which they find themselves [64, 65]. Evidence from climate research points to an association of collectivism and more climate-friendly actions that involve personal sacrifice [66]. Conversely, people from individualistic societies prioritize themselves and their own well-being; they are “first and foremost concerned about protecting their own systems of meaning” [28]. For example, in the early stages of the COVID-19 outbreak in the US, beaches in Florida were busy with spring breakers, socializing, and having fun [67]; college-aged healthy young Americans did not view the threat as personally relevant, and so death thoughts did not get activated.

Additionally, individualists may interpret recommended social contact avoidance as part of their normal pre-pandemic behavior. Following the diathesis-stress model and literature on adaptive coping for people with obsessive-compulsive disorders [68, 69], they would not substantially increase their already existing contact avoidance behavior. A pandemic may thus strengthen functional beliefs in individualistic societies, where people may experience a sense of competence as a result of their beliefs [70]. Taken together, I thus posit the following hypothesis about the influence of the individualism-collectivism bipolarity:

**H3**: A collectivistic culture is positively associated with increased social contact avoidance.

The way in which decisions are made within the family is an important aspect of its functioning [71]. Different family structures have been found to influence family practices related to decision making. There are clear cultural differences on whose opinion matters most and who has the final say [72]. How much responsibility adolescents carry depends on cultural values and expectations about power, autonomy, and cohesiveness within the family. A family structure offers social support, that is, the perception or experience of being loved, cared for, esteemed, and being part of a mutually supportive network. Extant research finds social support to be a causal contributor of health and well-being, because social support protects individuals from negative effects of stressful events (buffering hypothesis) [73], and gives a sense of predictability and stability in one’s life situation (direct effects hypothesis) [74]. The cohesiveness of the family structure refers to the degree of connectedness and emotional bonding between its members [75]. High cohesiveness is characterized by shared understanding, norms, and behaviors; “cohesive groups are better not only at spreading information, but also at generating normative, symbolic, and cultural structures that affect our behavior” [76]. Cohesion thus increases solidarity and loyalty, creates a sense of togetherness, and provides a source of emotional support in difficult situations [75]. Family cohesiveness specifically increases resilience of its members, that is, an increased ability to use cognitive and behavioral strategies to deal with stressful situations effectively: “Resilient communities develop environmental plans that allow people to feel safe when adversity hits” [77]. Within the context of a pandemic, I thus expect individuals from cohesive families to receive more social support and stay more at home. Formally:

**H4**: Family cohesiveness is positively associated with increased social contact avoidance.

Humane orientation means that a society “encourages and rewards individuals for being fair, altruistic, friendly, generous, caring, and kind to others” [78]. High humane orientation is associated with a rigid set of expectations, an emphasis on uniformity, and societal harmony [79]. Modern psychology regards the unity of humanity as each of its members caring for the well-being of all humanity, which is an expression of psychological maturity [80, 81]. Humans exhibit this social feeling [82] when they show for others “in general a deep feeling of identification, sympathy, and affection in spite of the occasional anger, impatience, or disgust […]. Because of this, they have a genuine desire to help the human race. It is as if they were all members of a single family” [83]. I thus hypothesize that people, who are genuinely concerned about others, are more likely to opt to stay at home during a pandemic. More formally:

**H5**: Humane orientation is positively associated with increased social contact avoidance.

Disadvantaged socioeconomic positions are widely associated with disease and mortality, “and there is no reason to think this will not be the case for the newly emerged coronavirus disease” [84]. A study from China indicates that severe cases of COVID-19 were more likely to be with agricultural workers and less likely to be with the self-employed [85]. Experience from the HIV epidemic in Africa shows that information and education campaigns can indeed alter people’s behavior [86]. Additionally, the optimal combination of educational campaign types oriented to decrease the infection rate by encouraging protective behavior (such as social distancing) or stimulating the infected to remove themselves from social activities (such as quarantining) has been studied mathematically [87]. The effectiveness of such health campaigns is obviously dependent on the recipients’ ability to comprehend and action on the message; a message needs to be understandable [88]. While health professionals are often reluctant to research the effectiveness of education interventions, the need for an evidence base is essential [89]. Thus, I posit the following hypothesis:

**H6**: Better education is positively associated with increased social contact avoidance.

### Time orientation

Time orientation has a long history in the philosophy and psychology literature; as such it is also a commonly cited aspect of national culture [90]. It can be thought of as a delineation of the past, present, and future [91, 92], as well as the difference between a monochronic and polychronic time system [93]. (Punctuality is a third aspect of time orientation [94], which is not relevant for this study.) While all societies need to cope with these three time phases, they differ in their focal ordering; that is, every culture has a preferred temporal perspective [95]. Past orientation emphasizes the maintenance or restoration of traditions, and present orientation is “timeless, traditionless, future-ignoring,” and pays attention to what is happening right now. Future orientation, on the other hand, stresses “planning for the future and hoping future is better than either the present or the past” [91]. It is also a characteristic of a well-balanced personality [96], and allows for planning, problem solving, and a delay of gratification. There is an affective aspect to the quality of the expected future, which influences individual behavior. Human motives are directed toward goals which may be attained in the future, and such goals comprise the achievement of certain ends or the avoidance of undesired events [97]. If we conceptualize a pandemic-free world as a desirable future state, then people can be expected to discontinue their traditional socializing behavior in the present. The degree to which they do that would depend on their level of future orientation. Extant research has, to this effect, linked levels of future orientation with individuals’ ability to set goals for future health, plan how to fulfill those goals, and execute plans accordingly [98]. To test this, I suggest the following hypothesis:

**H7**: Future orientation is positively associated with increased social contact avoidance.

Future orientation, as discussed above, is a multi-dimensional construct, and should always be considered together with other factors [97]. Notably, an extended future orientation does not necessarily indicate a well-adapted personality. An inverted U-shaped relationship exists between chronological age and life space extensity, which is the spatial and temporal part of the world that is relevant for an individual [99]. Older persons have a shorter future time perspective, simply because their remaining life expectancy is shorter [97]. The more time is perceived as finite, the less importance people attach to goals that expand their horizons, and the more importance they give to goals from which they derive immediate meaning [100]. During a pandemic and in countries where the average life expectancy is longer, I thus expect people to have more time to put their lives on hold and stay at home. More formally:

**H8**: Longer life expectancy is positively associated with increased social contact avoidance.

### Focus on accomplishments

From the perspective of the terror management health model, compliance to recommended health behaviors such as social distancing is more likely when it can be easily implemented, is immediately actionable, and thought to be effective for reducing the threat [28, 101]. However, social distancing may be more difficult to achieve in some social and cultural environments than in others. The cultural dimension of performance orientation is grounded on the idea of achieving societies [102], and reflects the extent to which a “society encourages and rewards group members for performance improvement and excellence” [78]. In particular, societies with high performance orientation value materialism [103], such that studies have found higher levels of innovative entrepreneurial activities in countries characterized by higher levels of performance orientation [104] and IPO activity [105]. Entrepreneurship is an activity that involves discovery, evaluation and exploitation of opportunities [106]; it thrives on interacting and networking with other people. Additionally, performance orientation has been characterized as “refer[ring] to a focus on looking smart and not looking dumb” [107]. Thus, I suggest that, in societies high on performance orientation, staying at home and avoiding social contacts is contrary to achieving performance-oriented goals:

**H9**: Performance orientation is associated with a reduction of social contact avoidance.

Assertiveness is the degree to which individuals in societies are assertive, tough, dominant, confrontational, and aggressive in their social relationships [108]. It is also positively related to the frequency and severity of hostile actions within cultures [109]. In a more favorable sense, aggressiveness is defined as being enterprising, or taking initiative [110]. Assertive societies reflect a belief in human perfectibility; they tend to take the view of dominance, reflecting the assumption that nature can be controlled and manipulated (doing orientation) [91]. “The proper thing for humans to do is to take charge and actively control their environment” [111]. Non-assertive societies, on the other hand, exhibit a being-orientation; implying a kind of fatalism, they assume that nature is powerful and humanity is deferential to it. “Because we cannot influence nature, we must become accepting and enjoy what we have. We must focus more on the here and now, […] and on acceptance of whatever comes.” During the pandemic, non-assertive people are therefore likely to “try to adapt to external realities rather than […] dominate some portion of the environment” [111]. Thus, I suggest the following hypothesis:

**H10**: Assertiveness is associated with a reduction of social contact avoidance.

Uncertainty avoidance is the extent to which people seek orderliness, consistency, structure, formalized procedures, and laws. By relying on established social norms, rituals, and bureaucratic practices, they strive to avoid uncertainty in their daily lives. And so, people in high uncertainty avoidance cultures actively seek to decrease the probability of unpredictable future events that could adversely affect the operation of an organization or society [78, 112]. In contrast, “ambiguity tolerance generalizes to the entire emotional and cognitive functioning of the individual,” which includes “interpersonal and social functioning, and problem solving behavior” [113]. Planning is a coping mechanism thought to control uncertainty. Inflexibility and resistance to change is associated with intolerance for ambiguity [114]. High levels of uncertainty avoidance also lead to a greater focus on short-term performance [112]. When the pandemic hit the world, the societal system became more entropic. In a tight culture, people are expected to conform to standard practices and thus lead highly structured lives [46, 115]. They find it difficult to suddenly change behavior, and stay at home. Thus, I hypothesize the following effect of uncertainty avoidance:

**H11**: Uncertainty avoidance is associated with a reduction of social contact avoidance.

A prior study within the US finds that the COVID-19 outbreak rate is positively linked to government spending (at the state level) and average household income (at the county level) [4], likely because the expansionary economic effect of public and household spending leads to more social interactions [116]. Dynamic economies rely on business interactions, and business interactions involve social contacts. Notably, “economic activity has as its ultimate end [in] consumption, not capital formation” [117]. However, intertwined social, economic, and human factors co-evolve and keep the future of a pandemic open-ended [118]. The detailed dynamic modeling of coupled systems is, however, out of today’s methodological reach [119]. Furthermore, the economic effects of a disaster usually only surface well after the disaster, that is, during the recovery phase [120, 121]. Because conducting business cannot be suddenly stopped or completely switched to remote interactions, I hypothesize that dynamic economies show a lesser extent of social contact avoidance:

**H12**: People in dynamic economies can be observed to engage less in social contact avoidance.

### Environmental psychology

People concerned about getting infected prioritize minimization of relatively costly detection errors [122], that is, “the error of missing a pathogenic cue and becoming infected is greater than that of perceiving pathogen threats where non actually exist” [30]. Consistent with this idea, pathogen threats influence how people perceive other individuals (e.g., people who are coughing) or other groups of individuals (e.g., people from a country with widespread infections). This mechanism has been confirmed in prior studies [32, 34]. Additionally, Wang and Ackerman (2019) proposed and confirmed with experiments that perceptions of social environments are also susceptible to threat management processes, such that dense social environments carry a higher risk of pathogen transmission than sparse environments, due to an increased potential for human contact. Across animals and humans, population density has been linked to increased likelihood of parasite and pathogen infections [123, 124]. Because people are generally aware of socially dense situations [30], being in such an environment increases arousal of self-protective concepts and behaviors [125]. Following environmental psychology in the conjecture of an interplay between human beings and their environment [126], I expect that people living in a densely populated country will engage in more social distancing behavior:

**H13**: The population density of a country is positively associated with increased social contact avoidance.

### Acceptance of rules

Power distance reflects the extent to which people accept and endorse authority, power differences, and status privileges. As an important aspect of culture, it has been related to a variety of behaviors in societies [127], such that power distance is positively related to conformity and agreeableness [128]. People with high power distance beliefs tend to enter into role-constrained interactions with authorities; they are more strongly regimented by the relative position of a superior [129]. In a dyadic relation, a more powerful other can restrict the available choices and make a person conform to role expectations [130, 131]. I thus expect individuals in high power distance cultures to more stringently follow rules and stay at home more often. More formally I posit:

**H14**: Power distance is positively associated with increased adherence to social contact avoidance rules.

## Materials and methods

**Fig 1:**
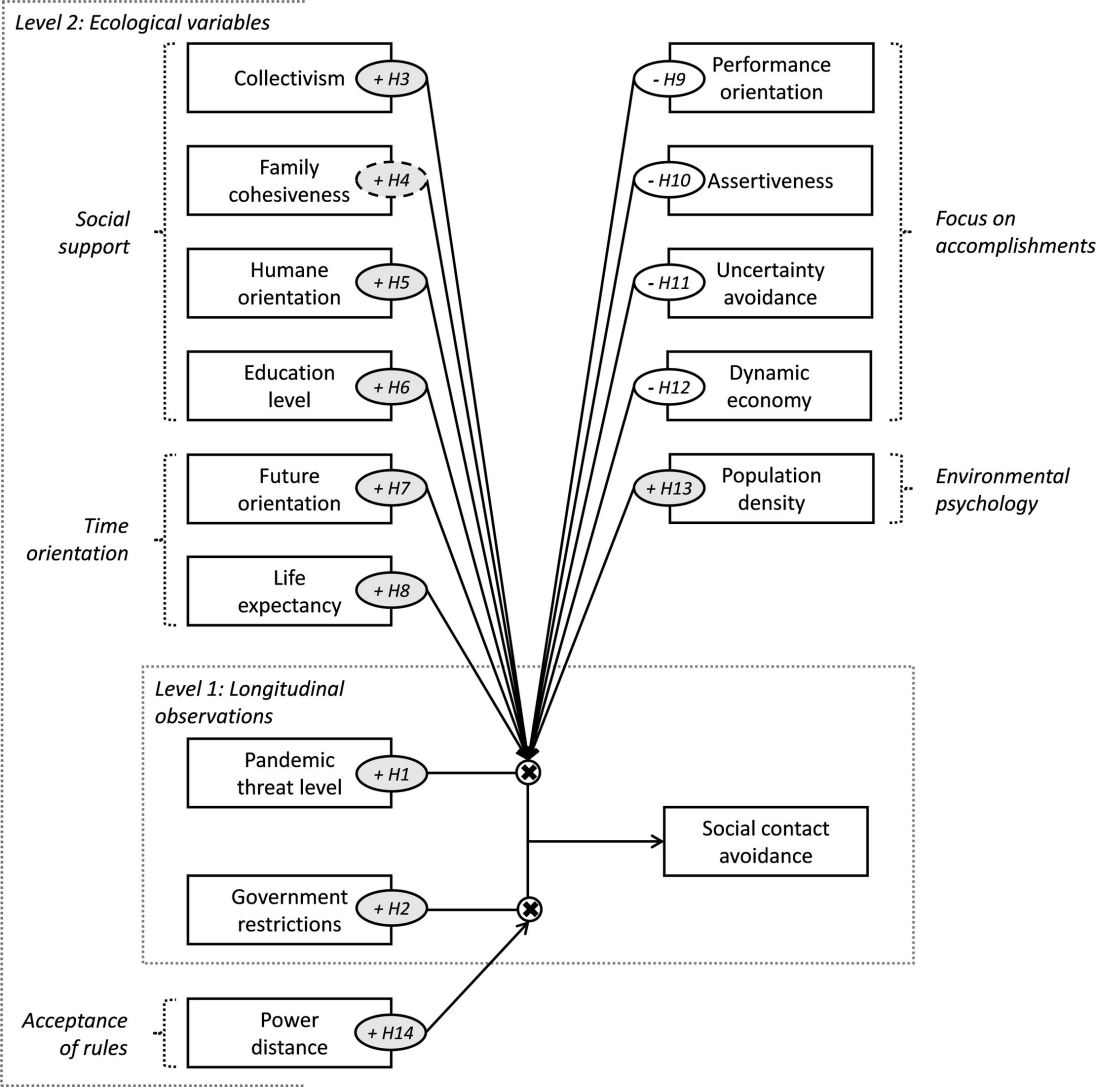
Research model. This diagram depicts the research model as a multilevel model with longitudinal country-level observations at level 1 and ecological country variables at level 2. Nested frames (grey dotted lines) indicate levels of sampling, boxes indicate variables, and arrows represent effects. Hypotheses are referenced in ellipses; the plus or minus sign in the grey or white ellipse indicate a positive or negative effect, respectively. A dashed ellipse (for H4) means that the effect is directionally indicative, but not statistically significant. Variables modelling the level-1 variance are not shown in the diagram, but are included in the statistical model.

As with much recent cross-cultural research in the psychological sciences, I employ contemporary geopolitical boundaries as a proxy for boundaries between different cultural populations. Consequently, geopolitical regions (countries) serve as the unit of my analysis. I now describe the research model (Fig 1) and the provenance of its variables (Table 1).

**Table 1:** Variables in the research model.

Variable	Abbr.	Level	Detail	Source
Longitudinal observations				
Social contact avoidance	MO7RESID	1 (dep.)	Google mobility data. Mobility change in residential areas, rolling 7-day average	[132]
Pandemic threat level	NEWDESMI	1	Our World in Data, Center for Systems Science and Engineering at John Hopkins University. Number of new deaths attributed to COVID-19 per million people, rolling 7-day average	[136,137]
Government restrictions	GOVSTRGI	1	Our World in Data, Oxford Coronavirus Government Response Tracker (OxCGRT). Strictness of lockdown style policies	[136,140,141]
Retail	MO7RTAIL	1 (het.)	Google mobility data. Mobility change in retail establishments (such as restaurants, cafes, shopping centers, museums, movie theaters), rolling 7-day average	[132]
Parks	MO7PARKS	1 (het.)	Google mobility data. Mobility change in parks (including beaches, public gardens), rolling 7-day average	[132]
Transit	MO7TRANS	1 (het.)	Google mobility data. Mobility change in public transport hubs, rolling 7-day average	[132]
Workplaces	MO7WORKP	1 (het.)	Google mobility data. Mobility change in workplaces, rolling 7-day average	[132]
Caseload	NEWCASSP	1 (het.)	Our World in Data, Johns Hopkins University. Number of new confirmed cases per million	[132]
Pandemic threat level x Government restrictions	M_NDESMI	1 (het.)	Interaction term; calculated	
Social support				
Collectivism	GCO1SV	2	GLOBE study. Institutional collectivism, societal practices	[151]
Family cohesiveness	GCO2SV	2	GLOBE study. In-group collectivism, societal values	[151]
Humane orientation	GHUMSV	2	GLOBE study. Humane orientation, societal values	[151]
Education level	HDISDG44	2	HDI. Education dimension (SDG 4.4). Country average for years of schooling in 2019.	[158]
Time orientation				
Future orientation	GFUTSP	2	GLOBE study. Future orientation, societal practices	[151]
Life expectancy	HDISDG3	2	HDI. Dimension SDG 3. Average life expectancy in year 2019.	[158]
Focus on accomplishments				
Performance orientation	GPERSV	2	GLOBE study. Performance orientation, societal values	[151]
Assertiveness	GASSSP	2	GLOBE study. Assertiveness, societal practices	[151]
Uncertainty avoidance	GUAVSP	2	GLOBE study. Uncertainty avoidance, societal practices	[151]
Dynamic economy	HDISDG85	2	HDI. Dimension SDG 8.5. Gross national income per capita, purchasing power parity	[158]
Environmental psychology				
Population density	POPUDENS	2	World Bank. World development indicator for population density; most recent year available	[136]
Acceptance of rules				
Power distance	GPOWSV	2	GLOBE study. Power distance, societal values	[151]
	GPOWSP	2	GLOBE study. power distance, societal practices	[151]

This table shows the provenance of the variables used in the research model Fig 1. The column Abbr. relates to the variable abbreviation used in the data files, which are available in the Online Appendix. dep. = dependent variable; het. = control variable to model heterogeneity of the dependent variable at level 1.

### Dependent variable

The study’s dependent longitudinal variable of interest is the extent of social contact avoidance during the COVID-19 pandemic, and I operationalize it as a mobility change in residential areas. Starting from February 15, 2020, Google publicly provided international mobility reports [132], which show how individuals across countries changed their visits and length of stay at different place categories. Previous studies have, for example, used this data to examine cross-cultural differences in panic buying behavior [133]. These mobility reports are calculated based on Google account holders who have opted-in to location history, so the data represents a sample of Google users. The data is available for 135 countries on a daily basis, relative to a baseline computed as the median value for the corresponding day of the week during the 5-week period from January 03 to February 06, 2020. Measuring mobility change relative to a normal value for that day of the week is helpful because people have different routines on different days of the week [134]. I use longitudinal data aggregated at the country level, from February 15, 2020 to January 10, 2021. A rolling seven-day average helps to reduce the amount of day-to-day variability in the raw data.

### Independent variables

There are two independent longitudinal variables in the model. First, I operationalize the pandemic’s threat level with the number of new deaths attributed to COVID-19 per million people living in that country. This is in line with terror management theory and reporting by popular media during the pandemic [135]. I source the variable from Our World in Data [136], which is based on the dashboard maintained by the Center for Systems Science and Engineering at Johns Hopkins University [137]. Because death figures on a given day do not necessarily show the number of deaths occurred, but the number of deaths reported on that day, I again use a rolling seven-day average [138]. Second, I operationalize the extent to which rules and regulations restricted social contacts with the composite index calculated by the Oxford Coronavirus Government Response Tracker (OxCGRT) project available in Our World in Data [136]. On any given day, the government stringency index “records the strictness of ‘lockdown style’ policies that primarily restrict people’s behavior” [139]; it is calculated as the mean score of nine metrics: school closures; workplace closures; cancellation of public events; restrictions on public gatherings; closures of public transport; stay-at-home requirements; public information campaigns; restrictions on internal movements; and international travel controls. On a scale of 0 to 100, a higher score indicates a stricter government response [140, 141].

Additionally, I use several longitudinal control variables to model heterogeneity of the dependent variable. First, I include a variable with the number of new confirmed cases per million people (as per Johns Hopkins University, Our World in Data [136]). Second, I add an interaction term between the two independent variables, pandemic threat level and government restrictions, because the strictness of lockdown-style policies (variable: government restrictions) goes hand in hand with the number of new deaths attributed to COVID-19 (variable: pandemic threat level). Lastly, Google’s international mobility reports provide similar baseline changes for places like parks (including beaches and public gardens), transit (public transport hubs), retail (restaurants, cafes, shopping centers, museums, movie theaters, etc.), and workplaces [132]. However, I do not use the baseline change for places related to grocery shopping (grocery markets, food shops/warehouses, farmers markets, drug stores, and pharmacies), because this variable is affected by panic buying and stockpiling in the initial phases of the pandemic [142–144].

### Independent ecological variables

I now move from the longitudinal variables to the independent ecological country-level variables. Because a culture is a “complex configuration of values” [145], I turn to the GLOBE (Global Leadership and Organizational Behavior Effectiveness Research Program) framework [146], which represents the most current data on cultural dimensions for a large number of countries [147]. This framework is particularly relevant to my purpose, because its societal dimensions conceptualize culture as what shapes differences in the collective practices and thinking of groups. The underlying items are at the targeted level of analysis, that is, the society [79], which makes the GLOBE dimensions ideal for developing theory in multilevel research [148]. Contrariwise, Hofstede’s framework [52] is more relevant to managerial practices, organizational patterns, executive leadership, and strategic decision-making [149, 150]. I accessed the GLOBE 2004 scores for the 62 GLOBE societies available at the project’s website [151]. Most GLOBE societies can be immediately matched with the countries in the Google mobility dataset, others according to usual practice in international business research [152], for example Canada (English-speaking) for Canada; England for UK; Germany-West for Germany; South Africa (white sample) for South Africa. At the end, I am left with 39 GLOBE societies (countries) that appear also in the Google mobility dataset. The descriptive statistics for this sample are comparable to those reported for the entire set of 62 countries [146]. Since the COVID-19 pandemic is an unprecedented event in people’s lifetime, I consider both societal as-is practice as well as to-be values for explaining human behavior [153]. Practices generally emphasize continuity, they are “a routinized type of behavior” [154], “taken for granted and often considered as part of the natural order of things” [155]. However, the changes to society that came along with COVID-19 had an intensity quite different from the gradual transformations which practice theorists tend to explore [156]. This justifies consideration of the value dimensions in addition to practices. To select the appropriate dimensions, I do not only look at their definitions [146], but at their composition at the item level [157]. This allows me to match the cultural variables as close as possible to the content domain of my hypotheses.

Specifically, in H3, I map collectivism to the societal values of GLOBE’s institutional collectivism (also referred to as collectivism 1); the items are about prioritizing group over individual goals. In H4, I operationalize family cohesiveness with the societal values of in-group collectivism (collectivism 2); the dimension is defined as “the degree to which individuals express (and should express) pride, loyalty, and cohesiveness in their organizations and families” [157]. For H5, I use the societal values of humane orientation; relevant items are about people being concerned about and sensitive to each other. The items speak vaguely of others as the targets of humane-oriented behavior. While it has been criticized that this leaves room for interpretation because the items do not differentiate between in- and out-group humane orientation [79], the intermingling of these two facets is ideal for the research topic of a pandemic, because pathogens spread from in- to out-group members through social contact. For H7, I use the social practices of future orientation, which capture the present versus future planning practices of societies. Conversely, the social values of future orientation reflect societal aspirations and preferences for planning [92]. For H9, I use the societal value of performance orientation, with items covering goal setting, performance, and performance effectiveness. For H10 and H11, the societal practices dimensions of assertiveness and uncertainty avoidance appear to be more appropriate than the values dimension, because the definitions of assertiveness and uncertainty avoidance describe actual and observable behaviors. For H14, both the societal practices as well as value dimensions of power distance are suitable for testing the hypothesis.

Regarding the other contextual variables, I use dimensions of the human development index (HDI) [158]. The HDI was created to emphasize that people and their capabilities are the ultimate criteria for assessing the development of a country, not economic growth alone. The HDI is frequently used in academic research as a contextual moderator [159]. For H6, I use the education dimension SDG4.4, which contains the 2019 country averages for mean years of schooling. For H8, dimension SDG3 measures average life expectancy for 2019. And for H12, I use a country’s gross national income per capita in purchasing power parity USD for 2017 (GNI; dimension SDG8.5) as a rough measure of annual income per person. Countries with a sizeable modern industrial sector have a much higher GNI per capita than countries that are less developed; dynamic economies increase the likelihood of attaining wealth through individual efforts [160]. Lastly, for the population density in H13, I use a world development indicator by the World Bank [136] for the most recent year available.

## Results

**Fig 2:**
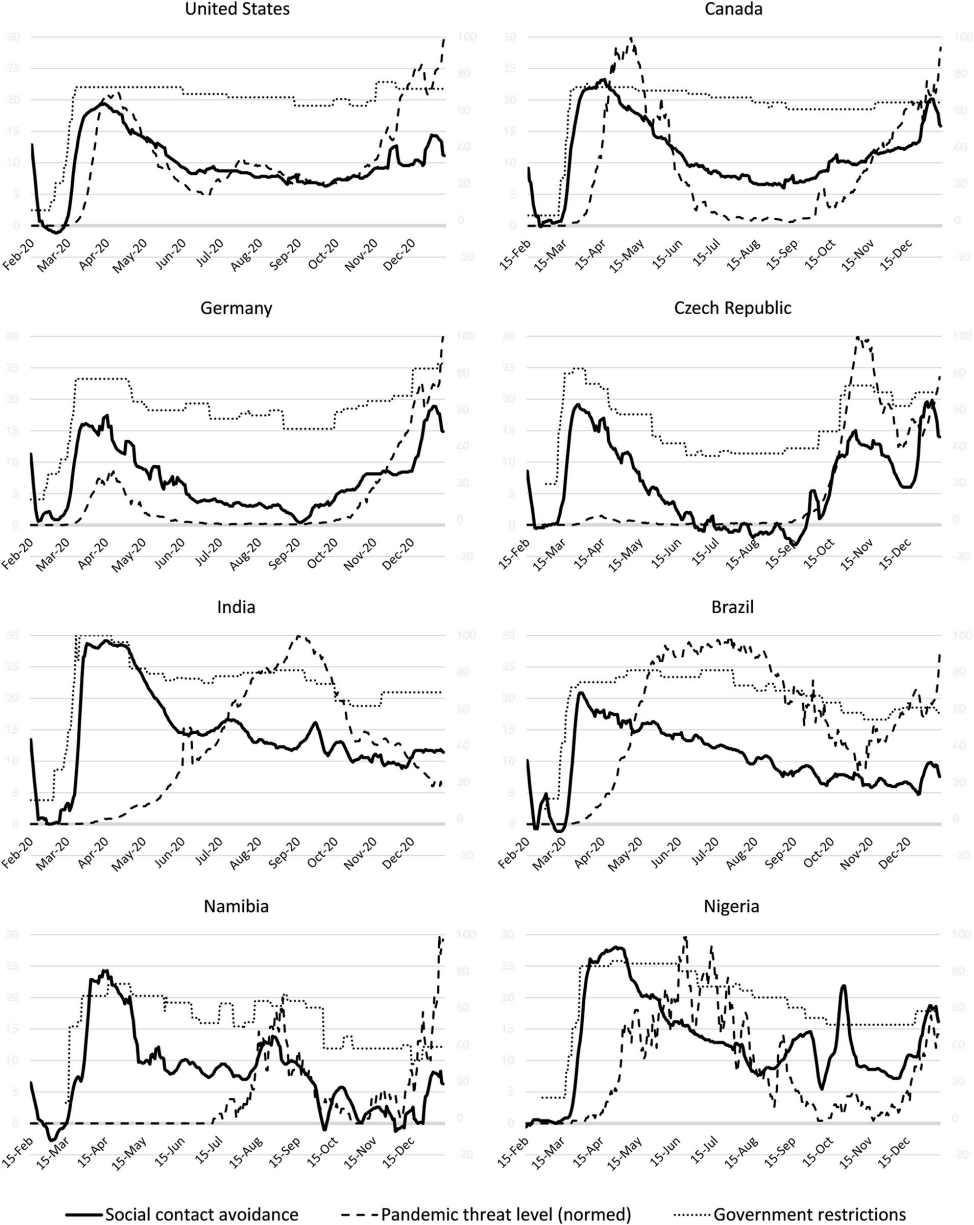
Social contact avoidance, pandemic threat level, and government restrictions. For eight countries from different geographies, this figure shows the extent of social contact avoidance (solid black line) from February 15, 2020 to January 10, 2021, alongside the pandemic threat level (dashed grey line), and government restrictions (dotted grey line). All three lines are on a separate scale; the line for the pandemic threat level is normed between zero and the country’s maximum threat level during the time period.

**Fig 3:**
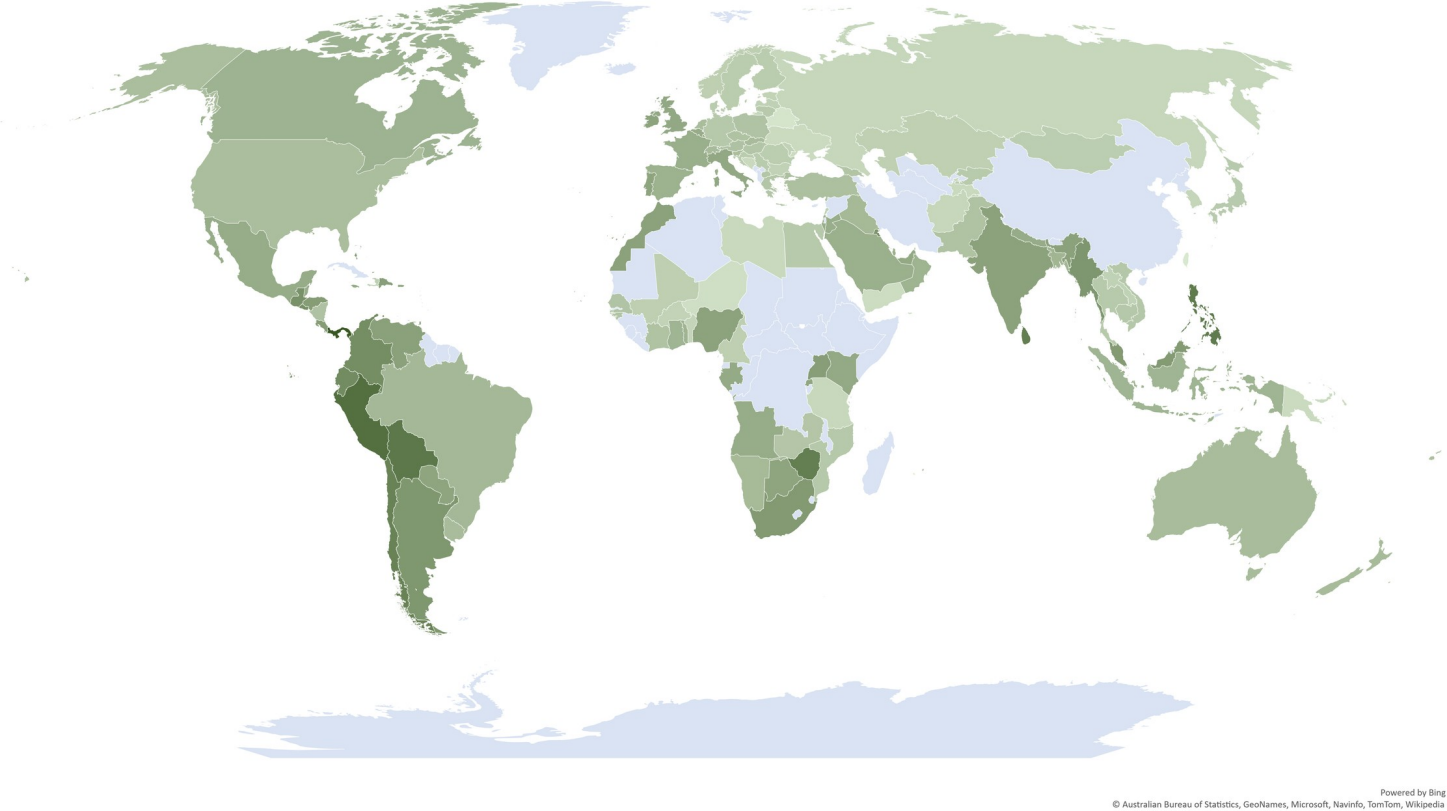
Social contact avoidance during the COVID-19 pandemic. Darker green colors signify countries with greater social contact avoidance during the COVID-19 pandemic. Lighter green colors stand for countries where social life went on more or less as usual. The measure used is the median plus one standard deviation of all residential mobility changes reported by Google for about 330 days from February 2020 to January 2021, against a baseline in January 2020. The data is available for 135 countries; no data is available for countries marked in light blue. (This is a chart created with Microsoft Excel^®^, based on data calculated by me in this present research; reproducing this map is thus considered to be fair use).

To begin with, I illustrate the longitudinal data. Fig 2 shows the extent of social contact avoidance from February 15, 2020 to January 10, 2021, alongside the pandemic threat level and government restrictions; Fig 3 illustrates average worldwide differences in social contact avoidance.

### Longitudinal multilevel research model

Next, I construct a longitudinal multilevel research model with the longitudinal dependent and independent variables together with the independent ecological variables listed in the previous section. At level 1 of the model (Fig 1), I am tracking information about social contact avoidance across time for the same panel of 135 countries with a total of 12399 datapoints, that is, there is a nesting structure in terms of multiple observations being nested within a single entity. When there is longitudinal data, the nested structure of the data must be accommodated in the analysis. A failure to do so would violate the independence assumption leading to biased standard error estimates and improper statistical inferences [161, 162]. At level 2, I can match the panel of 135 countries with 39 countries, for which all ecological data is available. I put the research model into operation with HLM 7.03 software [163], in which variables at level 1 (longitudinal observations within a country) are group-centered, and ecological variables at level 2 (country) are grand-centered [164, 165]. In the one-way random effects ANOVA null model, the within-group variability is σ^2^ = 48.456, and the between-group variability τ_0_ = 17.931. The proportion of variation in the outcome present between groups is ICC = 0.270, that is, about 27% of the variation in social contact avoidance is between countries. Thus, I build the following random intercepts fixed slopes model [166]:

Level 1: *Social contact avoidance*_*ti*_ *= π*_*0i*_ *+ π*_1i_*(Pandemic threat level_*ti*_*)*

*+ π*_2i_*(Government restrictions_*ti*_*) + e*_*ti*_;

Level 2: *π*_*0i*_ *= β*_*00*_ *+ r*_*0i*_;

π_1i_ = β_10_ + β_11_*(Uncertainty avoidance_i_) + β_12_*(Future orientation_i_)+ β_13_*(Assertiveness_i_)

+ β_14_*(Collectivism_i_) + β_15_*(Humane orientation_i_)+ β_16_*(Performance orientation_i_)

+ β_17_*(Family cohesiveness_i_) + β_18_*(Life expectancy_i_) + β_19_*(Education_i_)

+ β_110_*(Dynamic economy_i_) + β_111_*(Population density_i_);

*π*_*2i*_ *= β*_*20*_ *+ β*_21_*(Power distance practices_*i*_*) + β*_22_*(*Power distance values*_*i*_).

The ecological level-2 variables are not modelled to exert any influence on the intercept *π*_*0i*_ at level 1, because the level-1 variables pandemic threat level and government restrictions are baseline changes. Without changes in these variables, the level of contact avoidance would not change. And yet, I do not model a zero intercept so that the overall worldwide impact of the pandemic can be brought into the model. As compared to the null model, variation in social contact avoidance between countries has increased to more than 51% (ICC = 0.515; *r*_0_ = 19.784, *p* < 0.001). Estimated power to detect an effect size of 0.200 at a rather large expected effect size variability of 0.100 between countries is above 80%, calculated with Optimal Design software [167, 168]. Because multicollinearity is a serious issue in epidemiological studies [169], I calculate the correlation of the two independent variables to be 0.177, *p* < 0.001 (level 1, for all 38836 observations). I present the correlation matrix for the level-2 variables in Table 2; the average correlation is 0.287, and the highest 0.789 (between life expectancy and education), which is still below the typical cutoff of 0.8. Additionally, the automatic collinearity diagnostics by HLM 7.03 for the entire dataset do not detect any issues.

**Table 2:** Correlation matrix.

	11	12	13	14	15	16	17	18	19	110	111	21
11) Uncertainty avoidance	--											
12) Future orientation	0.765**	--										
13) Assertiveness	-0.094	0.089	--									
14) Collectivism	-0.320*	-0.261*	0.150	--								
15) Humane orientation	0.016	0.180	0.375**	0.087	--							
16) Performance orientation	-0.150	-0.101	0.111	0.448**	0.602**	--						
17) Family cohesiveness	-0.393**	-0.331*	-0.144	0.365**	0.278*	0.715**	--					
18) Life expectancy	0.251	0.164	-0.052	-0.181	-0.007	-0.188	-0.154	--				
19) Education	0.348**	0.321*	0.054	-0.645**	0.038	-0.279*	-0.285*	0.789**	--			
110) Dynamic economy	0.515**	0.436**	0.008	-0.334*	0.096	-0.171	-0.315*	0.706**	0.682**	--		
111) Population density	0.233	0.330*	0.154	-0.090	0.095	-0.074	-0.176	0.204**	0.117	0.300**	--	
21) Power distance practices	-0.512**	-0.444**	0.184	0.459**	0.462**	0.575**	0.500**	-0.256	-0.431**	-0.399**	-0.071	--
22) Power distance value	0.201	0.075	-0.163	-0.377**	-0.617**	-0.609**	-0.428**	-0.082	0.063	0.062	0.162	-0.557**

**. Correlation is significant at the 0.01 level

*. Correlation is significant at the 0.05 level.

This table provides the Pearson correlations between the ecological country variables at level 2 for 59 countries. Significance levels are for the two-tail test.

Modelling the level-1 variance to be a function of additional six variables improves the model fit (χ^2^ = 4290.325, *df* = 6, *p* < 0.001):

Var(*R*) = σ^2^ and log(σ^2^) = α_*0*_ + α_1_*(*Retail*) + α_2_*(*Parks*) + α_3_*(*Transit*)

+ α_4_*(*Workplaces*) + α_5_*(*Caseload*)

+ α_6_*(*Pandemic threat level × Government restrictions*)

With the assumption of heterogeneous σ^2^, differences between the countries in social contact avoidance still exist (*r*_0_ = 14.252, *p* < 0.001). Next, I consider the assumption of multivariate normality in the multilevel model. At level 2, a probability plot of the Mahalanobis distance and the expected values of the order statistic shows that the points are not substantially distanced from the reference line. The values for skewness and kurtosis of the residuals at level 1 are acceptable (skewness: 1.262, *se* = 0.022; kurtosis: 1.791; *se* = 0.044); a histogram shows that some non-normality exists in the left tail. Moreover, even severe non-normality in multi-level models would not cause the regression coefficients and associated standard errors to have a substantial bias [170].

### Hypothesis testing

The statistical results are in Table 3. As I am considering 14 prespecified hypotheses simultaneously in a confirmatory manner, I use the Holm-Bonferroni method for controlling the family-wise error rate [171, 172]. With the family-wise error rate prespecified at *α* = 0.05, I adjust the rejection criteria for each of the *k* = 1..14 hypotheses according to $\alpha^{*}=\frac{\alpha }{14+1-k}$, after sorting them according to their *p*-values. On average and across countries, the pandemic’s threat level and government restrictions have a positive amplifying effect on social contact avoidance, *β*_*10*_ *=* 0.572, *p* < 0.001 and *β*_*20*_ = 0.174, *p* < 0.001 respectively, which supports H1 and H2. Regarding the hypotheses on social support, collectivistic cultures amplify contact avoidance (*β*_*14*_ = 0.165, *p* = 0.004), in accordance with H3. However, the effect of family cohesiveness is only directionally informative, but not statistically significant (*β*_*17*_ = 0.099, *p* = 0.149); H4 can therefore not be supported. The influence of humane orientation is positive (*β*_*15*_ = 0.540; *p* < 0.001), in support of H5. Higher education levels are also associated with more contact avoidance (*β*_*19*_ = 0.086, *p* < 0.001), which supports H6. With respect to a focus on accomplishments, H9 to H12 are supported; performance orientation (*β*_*16*_ = -0.370, *p* < 0.001), assertiveness (*β*_*13*_ = -0.354, *p* < 0.001), uncertainty avoidance (*β*_*11*_ = -0.514, *p* < 0.001), and a dynamic economy (*β*_*110*_ = -1.30E-5, *p* < 0.001) all exhibit a statistically significant negative effect on social contact avoidance. Within the realm of environmental psychology, a country’s population density positively amplifies social contact avoidance, which supports H13 (*β*_*111*_ = 3.56E-4, *p* = 0.009). Lastly, a cultural tendency of power distance supports the influence of rules and regulations to increased social contact avoidance (*β*_*21*_ = 0.023, *p* < 0.001; *β*_*22*_ = 0.016, *p* = 0.005), which supports H14.

**Table 3:** Effects in the HLM contextual model.

**Panel A: Fixed effects**						
Fixed effect	Coefficient	*se*	*t*-ratio	*df*	*p*-value	α*
Mean country social contact avoidance, *π*_*0*_						
Intercept, *β*_*00*_	8.469	0.605	13.987	38	< 0.001	
For pandemic threat level slope, *π*_*1*_						
Intercept, *β*_*10*_	0.576	0.060	9.58	12345	< 0.001	0.003
Uncertainty avoidance, *β*_*11*_	-0.514	0.054	-9.607	12345	< 0.001	0.003
Future orientation, *β*_*12*_	0.582	0.061	9.549	12345	< 0.001	0.004
Assertiveness, *β*_*13*_	-0.355	0.069	-5.152	12345	< 0.001	0.004
Collectivism, *β*_*14*_	0.165	0.058	2.851	12345	0.004	0.012
Humane orientation, *β*_*15*_	0.540	0.075	7.242	12345	< 0.001	0.005
Performance orientation, *β*_*16*_	-0.370	0.040	-9.179	12345	< 0.001	0.005
Family cohesiveness, *β*_*17*_	0.099	0.069	1.445	12345	0.149	0.050
Life expectancy, *β*_*18*_	0.092	0.009	10.562	12345	< 0.001	0.006
Education, *β*_*19*_	0.087	0.022	3.896	12345	< 0.001	0.007
Dynamic economy, *β*_*110*_	-1.30E-05	2.00E-06	-5.436	12345	< 0.001	0.008
Population density, *β*_*111*_	3.56E-04	1.36E-04	2.611	12345	0.009	0.025
For government restrictions slope, *π*_*2*_						
Intercept, *β*_*20*_	0.174	0.002	87.647	12345	< 0.001	
Power distance practices, *β*_*21*_	0.024	0.005	5.128	12345	< 0.001	0.010
Power distance values, *β*_*22*_	0.017	0.006	2.791	12345	0.005	0.016
**Panel B: Random effects**						
Random effect	Std. dev.	Variance	*df*	χ^2^	*p*-value	
Mean country social contact avoidance, *r*_*0*_	3.775	14.252	38	16250.333	< 0.001	
**Panel C: Level-1 variance**						
Parameter	Coefficient	*se*	*Z*-ratio	*p*-value		
Intercept, α_*0*_	1.829	0.030	61.970	< 0.001		
Retail, α_*1*_	-0.030	0.001	-20.195	< 0.001		
Parks, α_*2*_	-0.006	0.000	-16.485	< 0.001		
Transit, α_*3*_	0.034	0.001	22.874	< 0.001		
Workplaces, α_*4*_	-0.054	0.002	-28.958	< 0.001		
Caseload, α_*5*_	-0.001	0.000	-11.993	< 0.001		
Pandemic threat level x government restrictions (interaction term), α_*6*_	-0.001	0.000	-17.394	< 0.001		

For the research model in Fig 1, this table shows the fixed effects (Panel A), random effects (Panel B), and the model for level-1 variance (Panel C). It is estimated as a longitudinal model with full maximum likelihood and 14 macro iterations using HLM 7.03 software. Because a heterogeneous σ^2^ is specified (Panel C), effect sizes of the coefficients cannot be computed in Panel A. After run-time deletion, there are 12399 level-1 records and 39 level-2 records. Standard errors are asymptotic. The α* is an adjusted α-value to control the family-wise error rate in large-scale hypothesis testing, according to the Holm-Bonferroni method. For all *p* < 0.001, the α* are sorted in order of variable occurrence, because HLM 7.03 does not return *p* with full decimals if *p* < 0.001. This does not affect hypothesis rejection.

### Robustness tests

I inform the above results with three robustness tests. First, this study conceptualizes culture at the national level to analyze implications of societal culture. While using cultural dimensions as averages of values and practices of individuals within societies does not represent ecological fallacy [173], the inability to collect individual-level cultural data can potentially introduce measurement error into the analysis. I therefore assess the robustness of the effects by excluding culturally more heterogeneous countries [174, 175], which are defined as being above the mean of a commonly used ethnic fractionalization index [176]. Note that a recent stream of research warns that cultural heterogeneity is not the same as ethnic fractionalization [177, 178]. However, in the absence of an established cultural heterogeneity index, I consider this approximation to be appropriate for a robustness test. Despite reduced power (now 33 countries), the direction of the effects and significance levels remain the same, with the following two exceptions: Family cohesiveness has a statistically significant negative effect (*β*_*17*_ = -0.716, *p* < 0.001), now leading to an outright rejection of H4. The negative effect of the dynamic economy is no longer statistically significant (*β*_*110*_ = -4.00E-6, *p* = 0.388), that is H12 can no longer be supported in the robustness test. Second, as explained in the methodology section, I consciously pick between societal practices and values aspects. Moreover, I omit gender egalitarianism, another cultural dimension of the GLOBE study, from the research model. There are reasons for limiting analyses to culture dimensions that are strongly tied to the research focus [179], yet arguably, “ignored cultural factors […] are contributing as much if not more to the observed effects” [180], and could explain further variance. Because value and practices dimensions of the GLOBE study are often strongly correlated, both negatively and positively [181], I iteratively add new dimensions to avoid multicollinearity issues, which, on the whole, proves the stability of the research model. Third, because it is nearly impossible to test for all possible confounding variables and because unexplained differences in the countries still exist (*u*_0_ = 14.252, *df* = 38, χ^2^ = 16250.332, *p* < 0.001), I quantify the potential impact of omitted variables on the independent variables [182, 183]. For instance, the necessary impact of such a confound for uncertainty avoidance *β*_*11*_ would be 0.815, that is, to invalidate the variable’s inference on the extent of social contact avoidance, another confounding variable would have to be correlated with both uncertainty avoidance and social contact avoidance at $\sqrt{0.815}=0.903$. Likewise, more than 78.444% of the countries would have to be replaced with countries for which the effect is zero. Table 4 shows these thresholds for all predictors in the HLM contextual model (Table 3), and, in summary, the effects are reasonably robust.

**Table 4:** Robustness of effects.

	Bias to invalidate inference		Confounding variable	
Effect	Threshold	Observations	Impact	Correlation
Pandemic threat level, *β*_*10*_	0.118	79.582%	0.070	0.265
Uncertainty avoidance, *β*_*11*_	0.111	78.444%	0.815	0.903
Future orientation, *β*_*12*_	0.125	78.495%	0.815	0.903
Assertiveness, *β*_*13*_	0.142	60.119%	0.545	0.738
Collectivism, *β*_*14*_	0.119	27.875%	0.185	0.430
Humane orientation, *β*_*15*_	0.154	71.502%	0.712	0.844
Performance orientation, *β*_*16*_	0.082	77.818%	0.806	0.898
Family cohesiveness, *β*_*17*_	0.142	30.073%	0.075	0.274
Life expectancy, *β*_*18*_	0.018	79.928%	0.835	0.914
Education, *β*_*19*_	0.045	48.115%	0.388	0.623
Dynamic economy, *β*_*110*_	< 0.001	68.433%	0.666	0.816
Population density, *β*_*111*_	< 0.001	21.615%	0.135	0.368
Government restrictions, *β*_*20*_	0.004	97.747%	0.608	0.780
Power distance practices, *β*_*21*_	0.010	57.254%	0.504	0.710
Power distance values, *β*_*22*_	0.012	27.582%	0.182	0.427

The robustness of the causal inferences is quantified for the effects in the HLM contextual model (Table 3), calculated with Konfound-It! 0.4.0 [183]. To invalidate the inference of each effect, the column observations shows the percentage of the estimate that would need to be due to bias. This is based on a threshold for statistical significance (α = 0.05), which is shown in column threshold. Additionally, the necessary impact of an omitted variable to invalidate an inference for a null hypothesis of zero effect is shown in column impact. That is, this omitted variable would need to be correlated with both the outcome as well as the predictor of interest at the level given in column correlation. At level 1, there are 8 covariates (including the variables for level-1 variance) and 12399 observations; at level 2, there are 11 covariates and 39 observations. In column threshold, the absolute value is given.

## Discussion

In this study, I aim to investigate the association of national culture and other contextual factors with social contact avoidance during 330 days of the COVID-19 outbreak in 2020. Studies using a large sample of countries are relatively rare in international business research, where typically only two to four countries are compared [184, 185]. Instead, at level 1 of the research model, I measure changes in social contact avoidance for 135 countries, against a pre-pandemic baseline; I source this data from Google’s publicly available international mobility reports. Longitudinal data for the pandemic’s threat level is from Johns Hopkins University, and the stringency of government restrictions is from the OxCGRT (both available via Our World in Data). At level 2, I collect information on national culture from the GLOBE study, HDI, and other World Bank indicators. This data is available for 39 out of the 135 countries.

### Theoretical implications

Building on terror management theory, the terror management health model, and the behavioral immune system, I extend knowledge in public health, epidemiology, and cross-cultural psychology by demonstrating the influence of national culture and contextual factors on social contact avoidance during the COVID-19 pandemic. Epidemics and infectious diseases are key to understanding historical change and societal development. In particular, I show the applicability of the terror management health model for the COVID-19 pandemic (H1), and how government restrictions were being followed (H2). The behavioral immune system managed the threat more actively in societies characterized by high social support (H3: collectivism; H5: humane orientation; H6: education level), even though the influence of family cohesiveness was not significant (H4). A related effect was evidenced for societies characterized by future time orientation (H7: future orientation; H8: life expectancy). Higher population density, a factor of environmental psychology, caused people to avoid social contacts more often (H13). Contrariwise, people in societies more focused on accomplishments did not change their behavior as much (H9: performance orientation; H10: assertiveness; H11: uncertainty avoidance; H12: dynamic economy).

### Policy and practical implications

Pandemics and epidemics are characterized by time-pressure and fear [186]. In such circumstances, it is difficult for professionals and the general public alike to put probabilities to the overall threat and individual harm–and act accordingly. My research suggests that taking cultural and contextual factors into account can help improve our understanding of human behavior during a pandemic. Although culture is increasingly considered in behavioral research, related theories might not fully apply to situations where a disruptive event occurs [142]. Most previous disasters were relatively local, which created only local reactions. With the COVID-19 pandemic, however and for the first time, a disaster had a direct, large-scale global impact on work and life [187], which raised the need for data-driven predictions [118]. Yet, the fundamental challenge was the nature of the pandemic, a classic wicked problem. Wicked problems are novel, unique, complex, evolving, and are based on incomplete, contradictory, and changing information [188]. The evolution of the pandemic was affected by many unknown unknowns related to the virus itself, human behaviors, reactions by authorities, intertwined political, economic, and societal issues in a global context [118]. In modeling, simulation, or forecasting, unknown unknowns are generally absent from current literature [189]. My current research reveals some of these cultural and contextual unknowns.

**Fig 4:**
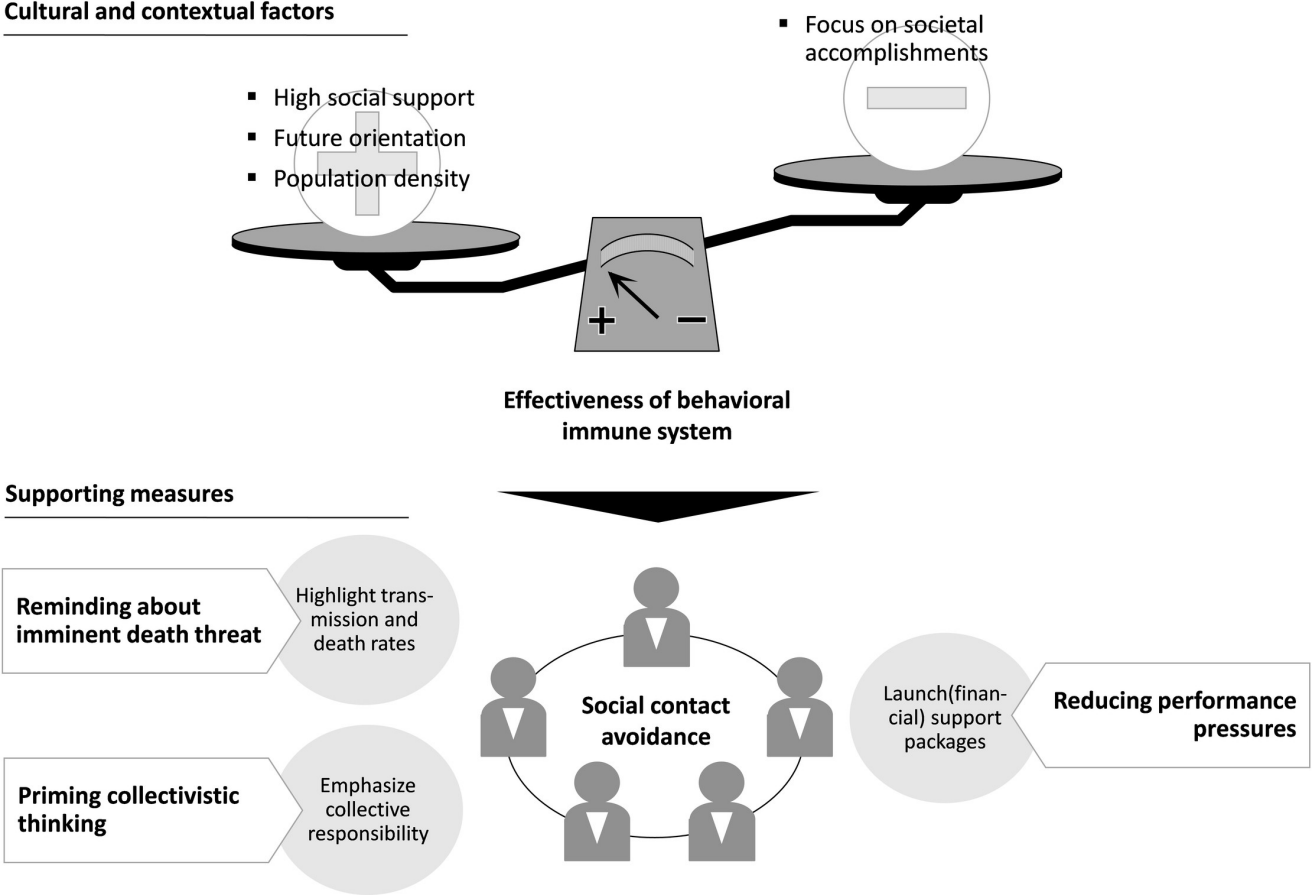
Policy and practical implications. This chart summarizes the study’s policy and practical implications. Because defensive behavior in a pandemic only makes sense at the group level, health-based communication needs to consider cultural and other contextual aspects.

It is well known in cancer-prevention research and the fear-appeal literature that health-based communication works best when thoughts of death are made painfully conscious [28]. I show in my empirical analysis that the pandemic’s perceived threat level is significantly associated with behavior change (H1). In a pandemic, communications aimed at changing public behavior should therefore include prominent reminders about the imminent death threat, for example by highlighting transmission and death rates. I further show that social contact avoidance is amplified in collectivistic societies (H3). Priming collectivistic thinking in a pandemic through targeted communication could thus increase social distancing. An effective health communication, in addition to stressing the threat, should thus also emphasize collective responsibility. It has been shown in previous research that temporarily priming a sense of collectivism is indeed possible [190]. Even though, deep-rooted cultural values are entwined at the deepest levels of human cognition. Value change is thus fraught with cognitive anxiety, and permanent value change is relatively rare [191]. However, changing human behavior during a pandemic is not so much a question of “manageability of culture,” but rather “one of manipulation and only during certain environmental conditions [including] crisis” [192]. Additionally, I show that a societal focus on accomplishments diminishes the threat effect on social contact avoidance (H9 to H12). Politics should therefore consider to take performance pressure away from people by timely launching and communicating various support packages. Taken together, these measures (summarized in Fig 4) should support social contact avoidance. Such intervention efforts are first and foremost communicative acts, which require policy makers “to understand human behavior through the prism of theory” [193]. From the perspective of the terror management health model, health behaviors function as defenses. But defensive behavior in a pandemic operates differently than, for example, cancer-risk behavior, because efforts to flatten the curve only make sense at the group level [28]. In contrast to the model’s traditional focus on decisions and behaviors affecting individual health, for a pandemic, cultural and institutional aspects need to be considered to understand sustained adaptation of behaviors to reduce one’s own risk, and the risk to others.

For domestic and international businesses alike, the COVID-19 pandemic created the need to understand in real-time the formative processes to the key decisions and strategies they had to take in order to response to newly-formed market pressures [13]. For doing that, understanding how and why their customers’ behavior has changed is a good starting point.

### Limitations and future research

Despite this study’s breadth in country coverage, it has some limitations in scope and methodology, which open up avenues for future research. First, the Google mobility reports “show how visits and length of stay at different places change compared to a baseline” [132]. Such “reporting [of] a percentage change from baseline” is frequently used in medical research, because it “gives the results in clinically relevant terms immediately accessible to patients and clinicians alike” [194]. Prior studies have used the mobility reports to analyze consumption patterns during the COVID-19 pandemic [133]. Yet, it is a controversial measure [195], which requires some attention: (a) Testing the relation between percentage change and baseline can yield spurious results, though I do not need to conduct such tests in my study; it is obvious from data visualization that nontrivial changes exist, and that such changes differ between countries. (b) The percentage change from baseline does not correct for imbalance between groups at baseline (chance effect) [196]. Thus, characteristics of smartphone and Google users in different countries could potentially influence observed social contact avoidance so that any differences are no longer due to different pandemic threat levels but due to characteristics of the Google users. Because the international mobility reports by Google are based on Google account holders who have opted-in to location history, they do not fully reflect a country’s population, but only a sub-sample of its Google users. I examine this potential bias at the country level, and find that national differences in smartphone penetration [197] are not correlated with this variable (*r* = 0.052; *p* = 0.611; 97 countries). In addition, some of the cultural variables used in my research model could influence the willingness of certain population groups within a country to opt-in to location history. As Google does not provide access to the underlying data, statistical tests of baseline characteristics are not possible. In comparative studies of health psychology, a degree of experimental control in the laboratory allows to obtain good data with a small number of participants [198], but in the current study, any kind of experimental control with respect to the dependent variable (social contact avoidance) is not possible. However, the mass location data produced by Google users via their smartphones [199] effectively averages all undesired effects, and ultimately permits a good degree of control at the ecological level. Additionally, I add control variables for heterogeneous level-1 variance into my research model to help adjust for any baseline imbalance [196]. As reported above (section Materials and Methods), these controls have helped to improve the model fit. (c) Because of Google’s baseline comparison to a period between January and February 2020, the data may reflect some changes in seasonal movement, rather than being fully explained by changes due to the pandemic [134]. For example, Fig 2 shows a seasonally higher residential mobility at the end of December 2020 (cf. India). While I can eliminate the amount of day-to-day variability by computing seven-day rolling averages, there is no way of eliminating seasonal variation. (d) Mobility categories are potentially defined differently across countries and regions, that is, direct comparisons can be misleading [132]. I have used a multilevel model in this study to counter these effects. Second, the attribution of deaths to specific causes is challenging under any circumstances, because health problems are often connected in multiplicative ways. While there are internationally established standards for death reporting, such as the cause-of-death classification [200], countries typically provide their own additional guidance to practitioners on the ground. Challenges due to limited testing means that the number of confirmed deaths may not be an accurate count of the number of actual deaths [138]. The multilevel model in this study again helps me to account for these country-level differences. Third, government response policies may vary at the subnational level. The government stringency index [136, 139] as an independent variable at level 1 shows the response level of the strictest sub-region. Furthermore, it is important to note that the index simply records the strictness of government policies, but does not measure or imply the response appropriateness or effectiveness. A higher score therefore does not necessarily mean that a country’s response is better than others lower on the index [141]. Future research could control for the appropriateness of government measures. Fourth, I leverage the GLOBE cultural dimensions [146] for examining culture’s role in social contact avoidance, because the GLOBE study contains societal dimensions for both cultural values and as-is practices. Additionally, the GLOBE uncertainty avoidance dimension focusses on rule orientation [201]. Other cultural frameworks are being used in areas such as cross-cultural psychology and international business, including the Hofstede framework [52] with its uncertainty avoidance focusing on stress [201]. Schwartz’s universal values [202], and Inglehart and Baker’s traditional versus secular and survival versus self-expression continua [203] are further frameworks. I also acknowledge that the notion of cultural dimensions has been widely debated, and may have inherent shortcomings. Fifth, I use longitudinal observations for about 330 days. It is nonetheless important to note that my approach is conditioned by the direction of influence. A dynamic relationship exists between the pandemic threat level and practices of social distancing, that is, the influence tends to run in both directions. While I use a one-directional model assuming that the threat level drives social distancing, the reverse direction of causal influence exists at the same time. Increased social contact avoidance may bring down the spread of the pathogen, and reduce the pandemic threat level. Voluntary social contact avoidance may also remove the necessity of imposed government restrictions. Furthermore, in the cognitive-behavioral model, safety behaviors are often a consequence of anxiety. But extant research also shows that health-related safety behaviors increase overall contamination of fear and health anxiety [204, 205], such that “individuals with high contamination fear perform more safety behaviors, which further increases contamination fear by enhancing the threat value” [9]. Future research could tease out circular relationships, and again examine differences between countries. Finally, I leverage secondary and ecological-level data alone to test the hypothesized relationships. Primary data would be needed to verify sociodemographic and individual-level cultural effects. A mixed-methods approach using both secondary and primary data would offer additional triangulation, and alleviate methodological concerns. As the COVID-19 pandemic was an unplanned event, given the speed of its outbreak, and considering travel restrictions, it was not feasible to set up international household panel studies. This opportunity has now passed, and post-hoc self-reports are rather weak for testing effects of disasters [16]. All things considered, I have confidence that my ecological analysis, which primarily relies on Google’s mobility reports, is suitable for testing the hypotheses.
